# Hemo-Therapin—A Notable Advance in Intravenous Therapy

**Published:** 1917-04

**Authors:** 


### Hemo-Therapin—A Notable Advance in Intravenous Therapy.

Long before Harvey made his epoch-making discoveries concerning the circulation of the blood it was recognized that this fluid played a role of paramount importance in the maintenance of healthy conditions of the body.

Many and various were the opinions held as to the manner, or way, in which the blood performed its functions, and though these were essentially crude and lacking in definiteness, it is interesting to note how strangely near the truth many of them were.

Following Harvey's discovery relative to the circulation, however, a decided impetus was given to the study of the blood, and it was soon established that the arteries constituted a great system of transportation canals through whose agency nutritive material was carried to the furthermost cells of the body, while the veins formed a sewage system for bringing waste and effete products to the organs of elimination. For a long time, therefore, the main functions of the blood were believed to be the distribution of nourishing pabula and the withdrawal of the products of combustion. But gradually, as a result of continued investigation, knowledge extended, and it was learned that under normal conditions the blood contains substances which have a very potent influence in protecting the body against the invasion of disease-producing organisms. In other words, that the blood through certain of its elements, both physiological and chemical, offers, in a state of health, a more or less effective defense against the germs of disease. Let these physiologic elements, however, become inactive, or the chemical substances become deficient, and the body straightway becomes an easy prey to pathogenic bacteria.

Still more recently it has been ascertained that in addition to substances which afford a more or less effective defense against germ invasion and attack, the blood also contains certain highly important agents which have the specific power of activating or regulating organic functions throughout the body. If these are present in normal quantity the various vital functions of the body are kept at normal activity. If, however, they are deficient or absent, the functions of the body fail to receive necessary stimulation, and the whole organism suffers.

Summed up, therefore, the blood is indeed "the life stream" of the body, since it conveys nourishment to the tissues, brings away waste material, provides defense against the attack of bacteria, and last but not least, supplies the vital substances which maintain functional activity of every important organ in the body.

For many years Dr. E. B. Witte, a prominent physician of Trenton, N. J., has devoted himself to the study of the blood. As a result of his researches, he early determined that when the blood is close to normal standards, the body is well nourished, the natural waste products are properly eliminated, an effective resistance is offered to the invasion of pathogenic bacteria, and the various functions of the body are kept normally active. But when, for one reason or another, the blood falls away from normal standards, the nutrition of the body suffers, the elimination of waste products is impaired, the resistance to germ attack is weakened, and the various functions of the body become sadly deranged and perverted. In other words, instead of the physiologic processes of the body being normally active, as soon as the blood is depreciated, they become depressed or deranged, with a loss of the physiologic harmony or equilibrium that constitutes a state of health.

Recognizing the relation of clinical conditions to these various phenomena, Dr. Witte reached the conclusion that the correction of many aberrant or diseased conditions depended on restoring the blood to as near to its normal state as possible. He accordingly applied himself especially to investigation of the chemistry of the blood, with the object of evolving a substance in liquid form that would so closely approximate normal blood in its essential chemical characteristics that when introduced into the circulation it would bring the blood nearer to the condition in which it exists in health.

It goes without saying, that the researches leading up to the discovery of a fluid meeting the foregoing conditions required many years of hard, painstaking labor. But at last Dr. Witte's studies and exhaustive experiments proved successful and a liquid was obtained which fulfilled the special conditions necessary to enable it to accomplish the results desired.

For several years this remedy, to which the name Hemo-Therapin has been given, has been subjected to the most extensive clinical tests, and the results it has achieved in a wide variety of ailments have verified and substantiated Dr. Witte's theories in a most conclusive and gratifying manner. Many cases of acute and chronic disease that have proven intractable to all other measures have responded promptly to one or two injections of Hemo-Therapin.

WHAT HEMO-THERAPIN IS.

Hemo-Therapin is a remedy in liquid form that in its essential chemical characteristics closely approximates those of normal blood serum. Introduced directly into the venous system it tends to restore the blood vitiated from any cause to a condition nearer to its original or healthy state. As a consequence of this sudden improvement in the chemical qualities of the blood, its physiologic activity seems to be materially increased. This must be so, for on no other basis is it possible to account for the pronounced results that are so uniformly observed, often following even a single injection of Hemo-Therapin.

Thus if the corpuscular elements are below normal, Hemo-Therapin promptly corrects this condition by stimulating the hematopoietic processes of the body. At the same time phagocytic activity is greatly augmented, the withdrawal of endogenous poisons is hastened and increased and functional activity of the various organs of the body markedly stimulated. Obviously a remedy able to accomplish the effects just mentioned may reasonably be expected to exert a pronounced influence on a wide variety of pathologic conditions.

This expectation is well borne out in actual experience, and Hemo-Therapin has proven one of the most valuable and dependable remedies at the command of the physician in the successful treatment of many acute and chronic affections.

Thus, in the acute affections, Hemo-Therapin may be confidently relied upon to increase the defensive forces of the body and rapidly control and overcome the infectious process. In erysipelas, septicemia, pyemia, the acute fevers, puerperal infection, furunculosis, carbuncles, malaria, acute rheumatism, pneumonia, typhoid fever, and in various skin diseases, such as eczema, psoriasis, herpes zoster, etc., the results have been prompt and gratifying.

In the more chronic ailments Hemo-Therapin is no less effective, although as may be anticipated it must be used over longer periods in order to produce the results desired. Thus diabetes, chronic Bright's disease, goiter, pulmonary tuberculosis, chronic rheumatism, the severe anemias, arterio-sclerosis, various nervous disorders, locomotor ataxia, varicose and indolent ulcers, and many other chronic diseases often respond to the influence of Hemo-Therapin when other remedies have proven powerless. Right here let it be said that no claim is made that Hemo-Therapin is an infallible panacea for every ill "that human flesh is heir to." Common sense teaches that when profound organic changes have taken place, with more or less complete obliteration of the original tissue of an organ or part of the body, temporary relief or palliation at best is all that can reasonably be expected. But Hemo-Therapin has done so much in cases that have been looked on as absolutely hopeless, that the practitioner is always justified in using Hemo-Therapin and giving his patient the benefit of the doubt. The results will often be surprising in the extreme.

Many other conditions might be enumerated in which Hemo-Therapin may be employed with beneficial results, but those mentioned will be sufficient to indicate the remarkable efficiency of Hemo-Therapin through its capacity to promote the elimination of tissue poisons and wastes, and stimulate the functional activity of cells throughout the body.

It is confidently believed that Hemo-Therapin represents a notable advance in the therapy of a great many diseases, and any physician who has some severe and intractable case on his hands that has resisted every form of treatment is urged to give Hemo-Therapin a careful trial. Every possible assistance will be given to any physician on request, and as happened so frequently in many other cases the results may prove gratifying in the extreme.

